# RF Energy Harvesting System Based on an Archimedean Spiral Antenna for Low-Power Sensor Applications

**DOI:** 10.3390/s19061318

**Published:** 2019-03-16

**Authors:** Antonio Alex-Amor, Ángel Palomares-Caballero, José M. Fernández-González, Pablo Padilla, David Marcos, Manuel Sierra-Castañer, Jaime Esteban

**Affiliations:** 1 Departamento de Lenguajes y Ciencias de la Información, Universidad de Málaga, Málaga 29071, Spain; angelpc@ugr.es; 2 Information Processing and Telecommunications Center, Universidad Politécnica de Madrid, Madrid 28040, Spain; jmfdez@gr.ssr.upm.es (J.M.F.-G.); dmarcosgon@gr.ssr.upm.es (D.M.); manuel.sierra@upm.es (M.S.-C.); jesteban@etc.upm.es (J.E.); 3 Departamento de Teoría de la Señal, Telemática y Comunicaciones, Universidad de Granada, Granada 18071, Spain; pablopadilla@ugr.es

**Keywords:** energy harvesting, Archimedean spiral antenna, Cockcroft-Walton multiplier, parasitic elements modeling, energy storage

## Abstract

This paper presents a radiofrequency (RF) energy harvesting system based on an ultrawideband Archimedean spiral antenna and a half-wave Cockcroft-Walton multiplier circuit. The antenna was proved to operate from 350 MHz to 16 GHz with an outstanding performance. With its use, radio spectrum measurements were carried out at the Telecommunication Engineering School (Universidad Politécnica de Madrid) to determine the power level of the ambient signals in two different scenarios: indoors and outdoors. Based on these measurements, a Cockcroft-Walton multiplier and a lumped element matching network are designed to operate at 800 MHz and 900 MHz frequency bands. To correct the frequency displacement in the circuit, a circuit model is presented that takes into account the different parasitic elements of the components and the PCB. With an input power of 0 dBm, the manufactured circuit shows a rectifying efficiency of 30%. Finally, a test is carried out with the full RF energy harvesting system to check its correct operation. Thus, the RF system is placed in front of a transmitting Vivaldi antenna at a distance of 50 cm. The storage capacitor has a charge of over 1.25 V, which is enough to run a temperature sensor placed as the load to be supplied. This demonstrates the validity of the RF energy harvesting system for low-power practical applications.

## 1. Introduction

There is no doubt that the appearance of the Internet has changed the conception of the world and our lives. Today, there are more devices connected to the Internet than people in the world. With this premise in mind, it seems logical to think that the Internet of Things (IoT) is called to be one of the major future revolutions [1]. Of course, the possibility of interconnecting embedded devices implies that the number of applications in which IoT has a place is practically unlimited.

**Figure 1:**
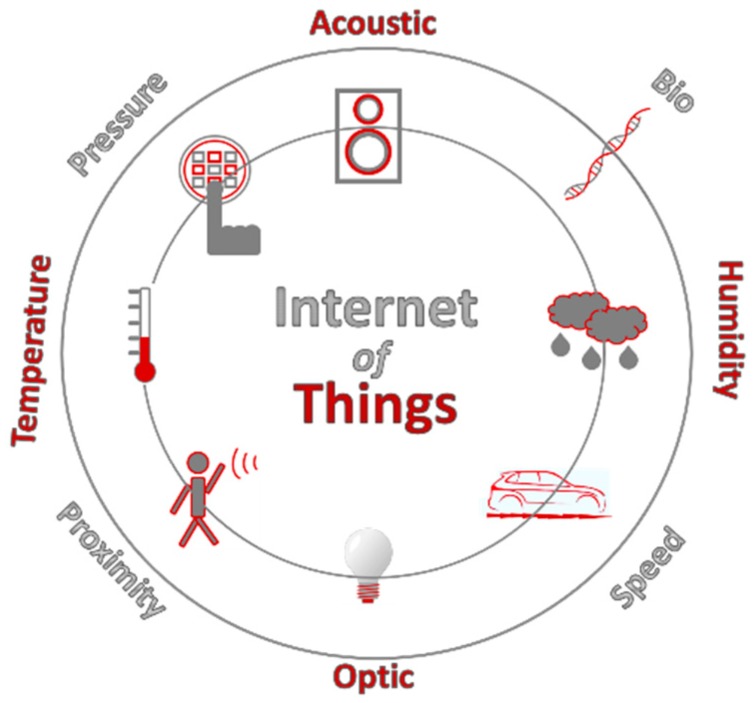
Some types of sensors used in IoT.

Sensors are going to play a crucial role in this scenario, as shown in Figure 1. They are a way of monitoring and extracting information from the environment that surround us. Evidently, IoT is clearly committed to the use of autonomous and self-sustainable sensors, such as those described in [2,3,4]. Power consumption is a key factor in this context, since it is difficult to combine self-sustainability and large operation times without the sensor being connected to the power grid. Nevertheless, the constant growth in the number of radiofrequency (RF) transmitters and early studies on the matter [5] have turned RF energy harvesting into a realistic and reliable power source. An extended overview of the topic can be found in [6].

In terms of power, almost all significant bands within the radiofrequency spectrum are located between 80 MHz and 5500 MHz. In particular, special attention should be paid to 800, 900, 1800, 2100, 2400, 3500 and 5200 MHz, where the mobile, WiFi and WiMAX frequency bands can be found. Another relevant power contribution comes from the FM band, which is typically located from 80 to 100 MHz in most countries. However, at these frequencies, the antenna would be too large.

Multiband antennas are typically used as the harvesting element. In general, they are easy to design because of their well-known characteristics. Thus, the triple-band antenna proposed in [7] is capable of operating at 2.1 GHz, 2.4–2.48 GHz, and 3.3–3.8 GHz. Frequently, slots or corrugations [8] are made in the structure to achieve multiple resonances or to reduce the wave propagation in a certain part of the antenna. However, this can be a double-edged sword, since the control of the mutual coupling between the slots themselves can be difficult [9]. On the other hand, wideband antennas can be harder to design, but in return, its broadband behavior ensures that the antenna will certainly cover the entire region of interest. Hence, an increasing number of authors are starting to use wideband antennas as harvesting elements. Wideband printed monopoles [10] and printed spirals [5,11] are the most common types of wideband antenna used in practice.

However, the current bottleneck of RF harvesting systems is the conditioning circuit, which is essentially in charge of rectifying the input signal. This is due to the intrinsic consumption of the diodes placed in the circuit. Since they are not ideal, they dissipate a high relative part of the total power for low input power levels. In consequence, the efficiency of a typical half-/full-wave rectifier, defined as the relation between the output DC power and the input RF power

(1) $$\eta =\frac{P_{out_{DC}}}{P_{in_{RF}}}$$

is typically under 25% for input powers of less than 1 mW (0 dBm). In [5], a half-wave rectifier circuit is implemented over an array of equiangular spirals. The results show that the efficiency of the rectifier ranges from 1% to 20%, depending on the input power. Some recent and very advanced studies [12] have achieved efficiencies of up to 74% for an input power of 1 mW. They externally feed the circuit in question and use the self-body biasing technique in a CMOS configuration to change the threshold voltage and turn on the transistor more quickly. Nevertheless, no schemes that have reached high efficiencies in the conditioning circuit make use of a true passive configuration; that is, they externally bias the elements of the circuit. The most used configuration for a conditioning circuit is the half-wave rectifier, but there are other, more complex configurations that are worth mentioning. For instance, the authors of [13] present a full-wave rectenna, formed by two concentric squared patches attached to a full-wave rectifier. The results show an efficiency in the rectifier of less than 34% for an input power of 1 mW. Another passive (not externally biased) configuration arises from multiplier circuits. They are capable of rectifying, but also elevating, the output voltage from the input RF signal. This is of great interest for our application, since the input voltage is on the order of hundreds of mV. In these terms, the authors of [14] introduce a 2.45 GHz rectenna based on a slotted patch antenna and a single-stage Cockcroft-Walton multiplier.

In this document, we present a radiofrequency energy harvesting system based on a ultrawideband Archimedean spiral antenna and a half-wave Cockcroft-Walton (HWCW) multiplier. The main aim of this work is to present a deep study of a complete RF harvesting system, paying attention to each individual block and to the entire system, as well as those elements, parameters and parasitic effects that are of importance in its design. The document is consequently organized as follows: Section 1 introduces the topic and reviews recent advances on the matter. Section 2 gives an overview of the system and the different stages that comprise it. Section 3 focuses on the design and manufacture of the Archimedean spiral antenna. Section 4 presents the matching and the conditioning circuits. Section 5 is reserved to test the operation of the complete harvesting system. Finally, conclusions are drawn in Section 6.

## 2. Global Design and Working Scheme

**Figure 2:**
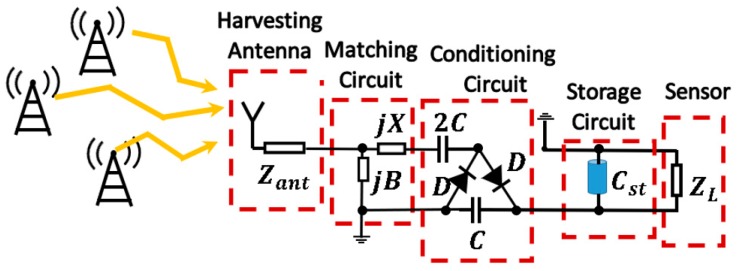
Full overview of the proposed RF energy harvesting system.

The complete working scheme is depicted in Figure 2. The RF energy harvesting system is formed of four stages: the first one is an Archimedean spiral antenna, an ultrawideband antenna that operates from 0.35 to 16 GHz. Even though the Archimedean spiral is a traditional antenna, its interesting properties and capabilities, such as the circular polarization, large bandwidth and omnidirectional pattern, have barely been exploited in RF energy harvesting. This cost-effective miniaturized design (FR4) acts as the harvesting element of the system, acquiring the available environmental power. Moreover, it is also utilized to characterize the power spectrum and to classify the most important spectral power peaks in real indoor and outdoor scenarios at the Telecommunication Engineering School of Universidad Politécnica de Madrid.

The second and third stages are the matching and conditioning circuits, respectively. The conditioning circuit is essentially in charge of rectifying the power harvested by the antenna. For this purpose, a Cockcroft-Walton multiplier has been used. As briefly discussed in Section 1, the multiplier circuit is also capable of elevating the output voltage apart from rectifying. This is a key point in energy harvesting, as the increase in the input voltage makes the acquired energy more manageable. Then, the matching circuit is designed to maximize the power transfer between the spiral antenna and the conditioning circuit. This stage is implemented through an *L* matching network with lumped elements. As will be seen, the parasitic elements of the circuit play an important role in the design of conditioning and matching circuits. They have to be taken into account and to be properly modeled. Otherwise, the efficiency of the circuit would be considerable reduced. Thus, the most harmful parasitics are identified in the circuit, and a circuit model is created to correct the frequency shift they produce.

The fourth stage is the storage circuit. It lays up the power previously acquired by the antenna and subsequently processed by the matching and conditioning circuits. It is formed by a high-capacity shunt capacitor, $C_{st}$, which feeds the sensor appropriately.

Multiple tests were carried out to check the correct operation of each stage separately. Additionally, the four stages were connected one to each other and the full system was tested in the laboratory in a real scenario. Unlike the vast majority of studies, we used a real sensor—a temperature sensor—as the load to be supplied. We proved that the sensor is perfectly operational if the antenna is able to collect at least −4 dBm, which is less than the power demonstrated to be acquired by the spiral antenna in Figure 8.

## 3. Archimedean Spiral Antenna

Spiral antennas are generally classified as frequency-independent antennas [15]. This kind of antenna has the property of maintaining some of their radiation parameters constant, such as the impedance. Thus, they are extensively used in applications where a large bandwidth is required. Their constant impedance along the frequency facilitates the design of the multiplier circuit, since the circuit dependence on the antenna impedance is eliminated. The two spiral-shape antennas most typically used in practice are the Archimedean spiral [11,16] and the equiangular spiral [17], both circularly polarized. The design of the harvesting element will be based on the Archimedean spiral antenna due to its simpler geometry.

### 3.1. Design and Simulation

**Figure 3:**
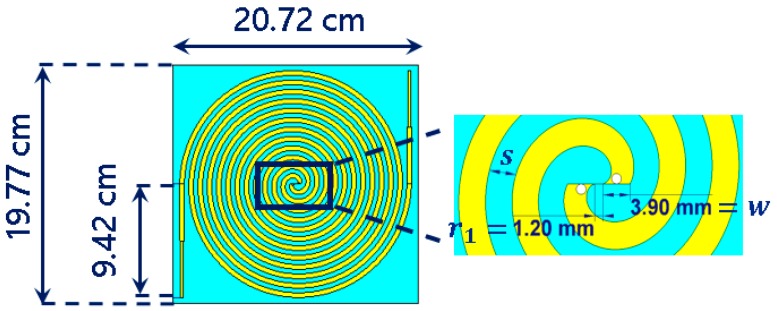
Archimedean spiral antenna.

Miniaturization plays an important role in the design of the harvesting element. Thus, the dimensions of the Archimedean spiral can be reduced without degrading the operation of the antenna. Multiple miniaturization techniques have been studied to date. The most common ones try to modify either the feed line [18] or the ground plane [19] in the antenna. Furthermore, some newer and more complex techniques use fractal geometries (Koch curve) [20] to increase the electrical length of the antenna. In our design (Figure 3), we simply propose extending the arms of the spiral in a straight line to the end of the antenna. The studies carried out in [11] demonstrate that it is preferable to put an impedance step in the center of the arm extension. Thus, current reflections are softened, and a higher bandwidth can be reached.

**Figure 4:**
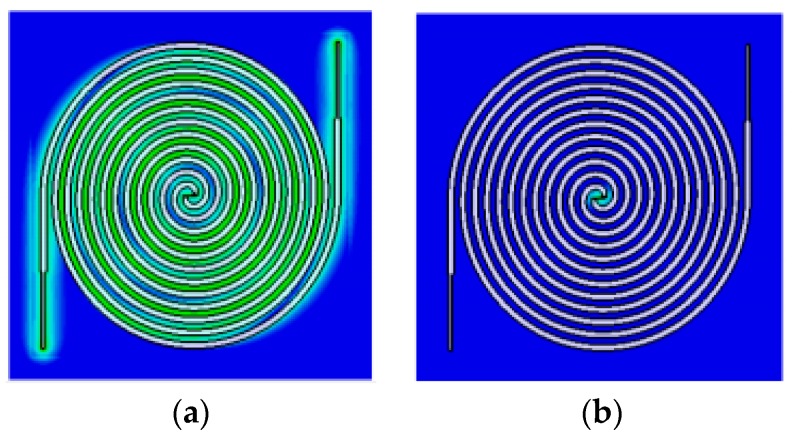
Current distribution over the Archimedean spiral antenna at 0.35 GHz (**a**) and 10 GHz (**b**).

**Figure 5:**
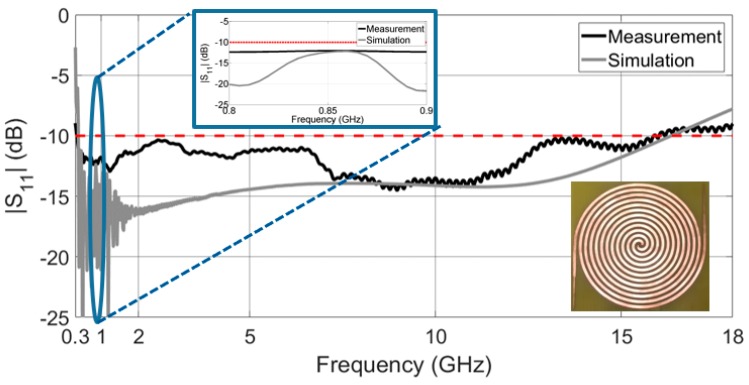
Measured and simulated reflection coefficient (with respect to 188 Ω) and an inset that zooms the 800–900 MHz band.

Some researches [5,9] have pointed out that the surface current distribution over the Archimedean spiral opens to the end of the antenna at the lowest frequencies and moves to its center at the highest operation frequencies. This fact is visualized in Figure 4. This particular current distribution is frequently used as a rule of thumb in order to initially estimate dimensions of the antenna [9]. The number of turns in the spiral, *N*, is directly related with the lower cutoff frequency. The higher *N* is, the lower the lower cutoff frequency, as the antenna is bigger. On the other hand, the inner radius $r_{1}$ mainly fixes the upper cutoff frequency. The antenna must operate from 400 MHz to up to 5 GHz, in order to capture all frequency bands of interest. Thus, the number of turns *N* and the inner radius $r_{1}$ are chosen to be N=6 and $r_{1}$ = 1.20 mm, so the antenna has dimensions of 20.72 cm × 19.77 cm. A low-cost FR4 substrate ($\varepsilon_{r}$ = 4.7, tan δ = 0.014 @1 MHz) is used to give mechanical rigidity to the structure and to ease the subsequent placement of a rectifier circuit. The design of a theoretically infinite self-complementary Archimedean spiral (the metallic strip width, *w*, is equal to the separation between two adjacent strips, *s*) ensures that the antenna impedance is constant along the frequency [15]. Its value is calculated according to the Babinet’s principle [9] and results in 188 Ω. Since the size of the antenna cannot be infinite in practice, the values of *w* and *s* play an important role in controlling the bandwidth of the antenna. An optimization process is carried in commercial software CST Microwave Studio to determine the value of the strip width. For a value of *w* = *s* = 3.9 mm, the antenna is perfectly matched from 350 MHz to 16 GHz, as Figure 5 shows. A ripple is observed at frequencies below 1.5 GHz, since the antenna becomes electrically short and the impedance starts to move from its constant value of 188 Ω. However, note that the ripple is mostly below −15 dB. This implies a minimal variation of the impedance, which stands out in Figure 5 due to the decibel scale in the *y* axis.

**Figure 6:**
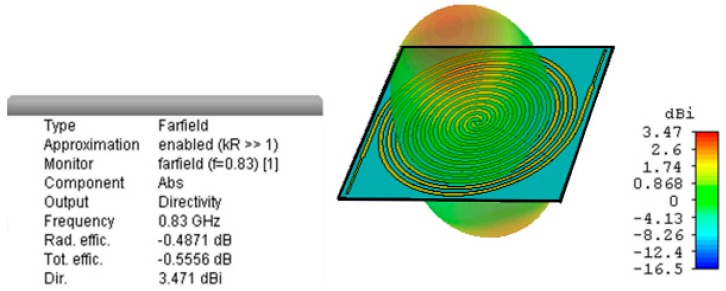
3D farfield radiation pattern at 830 MHz.

Figure 6 shows the farfield radiation pattern at 830 MHz of the miniaturized Archimedean spiral. The antenna presents an omnidirectional radiation pattern, as no ground plane is used. It radiates perpendicularly to the plane of the antenna, with two opposite symmetrical main lobes. The omnidirectional pattern is of great usefulness in RF energy harvesting, since we do not know a priori the direction of arrival of the incoming signals. On the other hand, directional antennas [21] should be used if the location of the main radiating source is known. Figure 6 reveals that the antenna efficiency is quite elevated, at 88%. This way, we ensure the antenna does not waste part of the incoming energy. The Archimedean antenna is circularly polarized, as are the vast majority of spiral-shaped antennas. This is very useful in radiofrequency energy harvesting, since the polarization of the incoming signals is unknown and a circularly polarized antenna minimizes depolarization losses. The simulation results in [11] demonstrate that the Archimedean spiral antenna presents circular polarization in a wide range of elevation angles at all relevant frequency bands but under 500 MHz, where it remains elliptically polarized.

### 3.2. Prototype

**Figure 7:**
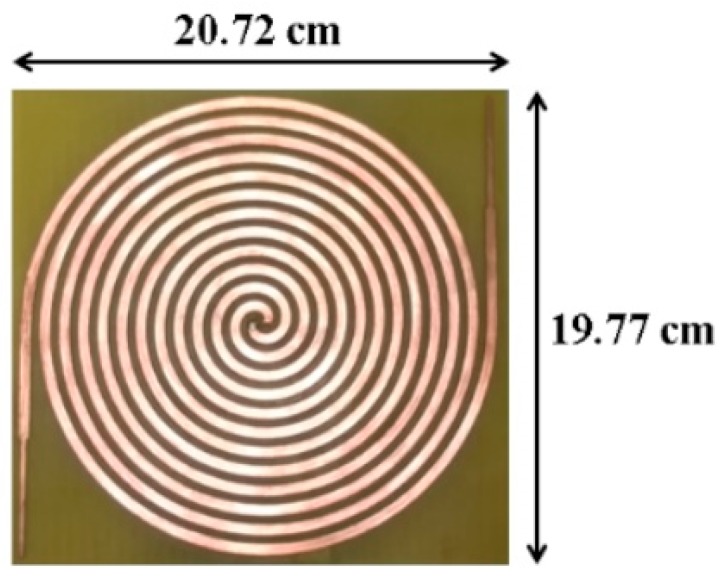
Prototype of the manufactured Archimedean spiral antenna.

To test the correct performance of the antenna, a prototype of the Archimedean spiral is manufactured with a LPKF ProtoMat S100 milling machine, as shown in Figure 7. As discussed before, both arms are extended with an impedance step to the end of the FR4 substrate to improve the bandwidth of the antenna. The reflection coefficient is then measured in the laboratory and compared with the simulated one. Both results are depicted in Figure 5. With a bandwidth exceeding 15 GHz, measurements prove that the antenna covers most frequency bands of interest. It is perfectly matched from 0.35 GHz to 16 GHz, showing an ultrawideband performance.

The antenna is sensitive to small variations in the metallic strip width *w*, in the separation between two adjacent strips *s*, and in the inner radius $r_{1}$. Thus, although the spiral has an outstanding bandwidth, there exist differences between the simulated and the measured curves, mainly caused during the manufacturing process. Beyond that, the upper and lower cutoff frequencies (16 GHz and 350 MHz, respectively) coincide perfectly in both graphs of Figure 5.

### 3.3. Harvested Power

Once it has been demonstrated that the antenna is able to properly operate as an ultrawideband harvesting element, a realistic measurement of the harvested power must be done. The components of the conditioning circuit (nonlinear circuit) are subsequently estimated according to the frequency bands with the highest contribution of spectral power. Thus, the spiral antenna is connected to a N9020A MXA signal analyzer and tested in two different scenarios: indoor and outdoor. Both scenarios take place in the facilities of the Telecommunication Engineering School, Universidad Politécnica de Madrid (UPM), Madrid, Spain.

**Figure 8:**
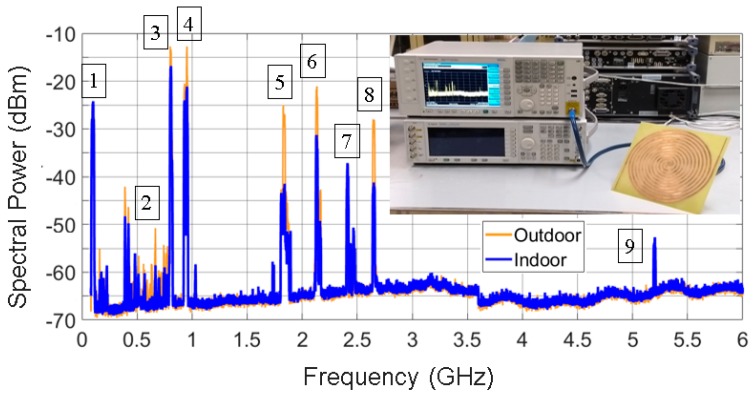
Spectral power acquired by the Archimedean spiral antenna in outdoor and indoor scenarios (resolution bandwidth: 1 MHz). The most relevant frequency bands are remarked: 1—FM, 2—DTT, 3—LTE-800, 4—GSM-900, 5—GSM-1800, 6—LTE-2100, 7—WiFi, 8—LTE-2600, 9—WiFi.

**Figure 9:**
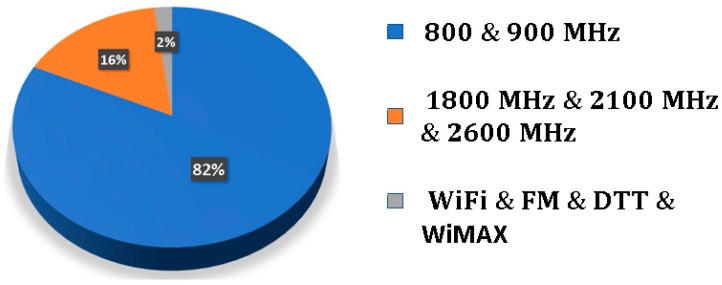
Distribution of the acquired spectral power outdoors.

Figure 8 presents the spectral power measured indoors and outdoors, and the measurement setup for the Archimedean spiral antenna. The frequency bands with major power contributions in the radio spectrum are the mobile assignment bands (marks 3,4,5,6,8 in Figure 8). Concretely, this accounts for 98% of the total power outdoors, as Figure 9 shows. It can be seen that the remaining 2% is contributed by the FM, WiFi, DTT and WiMAX bands. Thus, the power that comes from WiFi bands (marks 7 and 9 in Figure 8) is almost negligible in this particular case. It is very useful to know that 82% of the harvested power is located in the 800 MHz and 900 MHz bands. As a result of this, future antennas can be designed to uniquely operate at these frequencies. Thus, no significant amount of power will be lost, and the design can be greatly simplified.

From 5.2 GHz onwards, at present, only satellite and military applications can be found. However, the antenna gain is so reduced that no significant power contribution is obtained. As a result of this, a −65 dBm noise level is captured over a large frequency range (5.2 GHz–16 GHz). Nevertheless, the contribution of the noise to the total harvested power is negligible. This fact indicates that it is not necessary to design an omnidirectional wideband antenna with an upper cutoff frequency above 5.2 GHz.

The measured spectral power is higher outdoors than indoors, as can be noticed in Figure 8. This is reasonable. The higher spectral contribution comes from outdoor radio sources (base stations), and the signals are attenuated by the walls, windows, and the buildings themselves when measuring indoors. That is why the power level of FM band is almost identical indoors and outdoors, since 100-MHz signals penetrate walls more easily than higher frequency signals. Hence, the attenuation suffered is lower.

Finally, the total harvested power is calculated from Figure 8 by integrating the acquired spectral power (with a resolution bandwidth of 1 MHz) in the displayed bandwidth (0.1–6 GHz). This power is the actual available power between the arms of the antenna. Therefore, it is the power that is going to be delivered to the conditioning circuit. This is determined to be −3.19 dBm (480 μW) indoors and 1.86 dBm (1535 μW) outdoors. Both values are sufficient to feed low-power sensors, which generally consume less than 30 μW [6]. Evidently, not all of the collected power will be available for the sensor. Part of it will be lost in the conditioning circuit.

### 3.4. Circuit Model of the Archimedean Spiral Antenna

As previously discussed in Figure 9, the most part of the acquired power comes from the mobile frequency bands. In particular, the greatest power contribution comes from 800 MHz and 900 MHz. Thus, this particular distribution of the spectral power makes it possible to focus the design of the rectifying circuit at these particular frequencies. Additionally, the design of a wideband matching circuit is an incredibly complex task.

**Figure 10:**
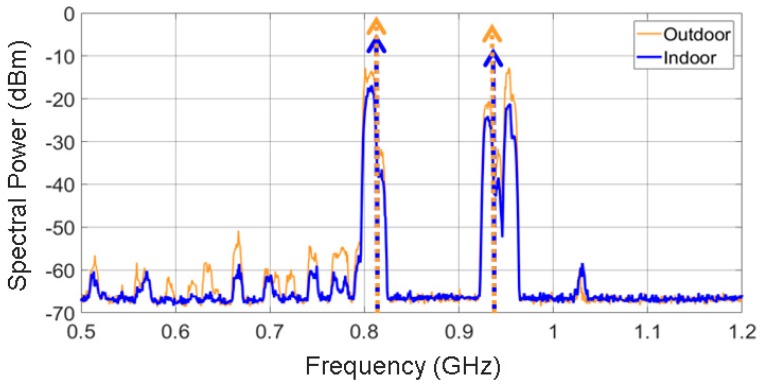
Zoom on the 800/900 MHz frequency bands over the spectrum acquired by the Archimedean spiral antenna (resolution bandwidth: 1 MHz).

**Figure 11:**
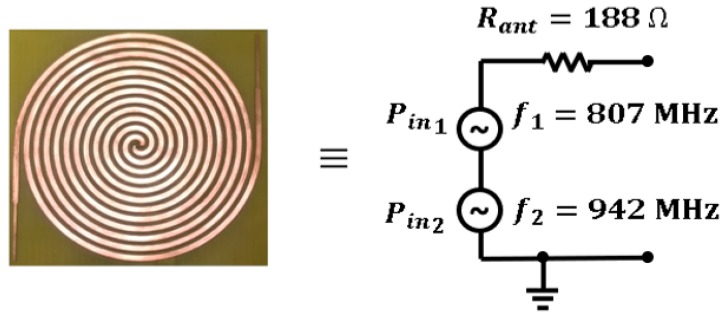
Circuit model of the Archimedean spiral antenna.

A zoom on the spectral power in the 800 and 900 MHz frequency bands is presented in Figure 10. There is no straightforward manner to obtain the analytical expression of both waveforms, due to their intricate shapes. Since the bandwidth of both waveforms is narrow enough, they can be modeled as two delta functions centered at 807 and 942 MHz, respectively. The power of both delta functions is obtained by integrating the spectral power of each frequency band. Hence, the waveform centered at 807 MHz is integrated from 770 to 820 MHz, obtaining a value of $P_{in1}=-5.80$ dBm indoors and $P_{in1}=-1.46$ dBm outdoors. Afterwards, the waveform centered at 942 MHz is integrated from 920 to 970 MHz, obtaining a value of $P_{in2}=\;-9.20$ dBm and $P_{in2}=-2.34$ dBm indoors and outdoors, respectively. In short, the spiral antenna is modeled, via the acquisition of the power spectrum in the laboratory, as two delta functions. As depicted in Figure 11, these functions are circuitally represented as two sinusoidal series generators with a source impedance of 188 Ω (the spiral impedance).

## 4. Circuit

This section is focused on the discussion of the matching and the conditioning circuits. The matching circuit is implemented via an *L* matching network, while the conditioning circuit makes use of a half-wave Cockcroft-Walton (HWCW) multiplier. Unlike a simple rectifier circuit, the Cockcroft-Walton multiplier is also capable of elevating the output voltage while rectifying the input signal [22]. This is of great interest in energy harvesting applications, where the input voltages are very low. The HWCW is a multiplier circuit widely used in power electronics [23] in the generation of high DC voltages. Therefore, it has typically been implemented in particle accelerators [24], X-ray machines [25], and cathode ray tube televisions [26].

**Figure 12:**
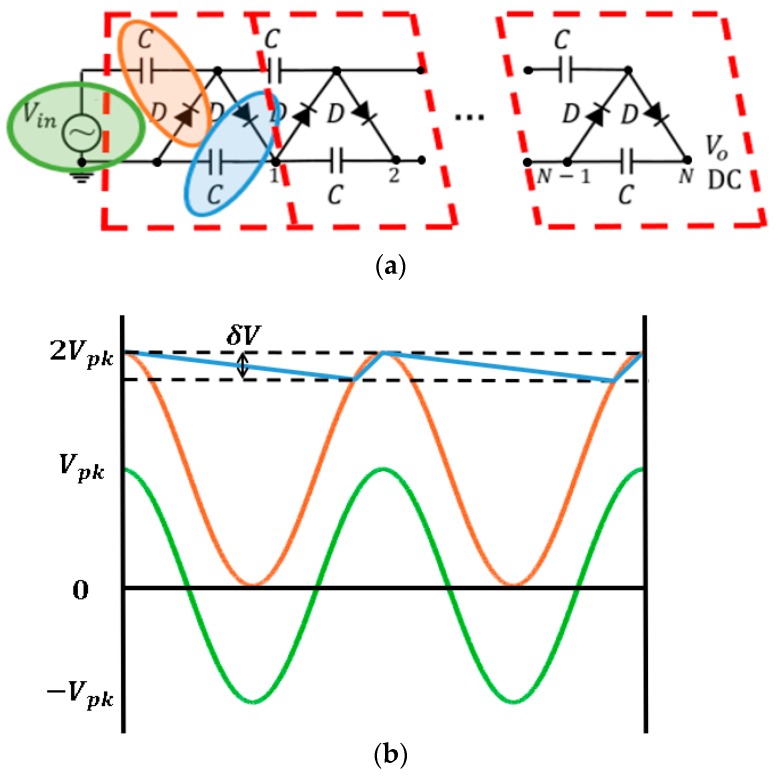
*N*-stage half-wave Cockcroft-Walton multiplier (**a**) and its working principle (**b**).

Two circuits constitute a single stage of a HWCW multiplier [27]. The first circuit (marked in orange in Figure 12a) is a positive diode clamping configuration, also known as Villard circuit [23]. It is responsible of elevating the DC level of the AC input waveform. The second circuit (marked in blue in Figure 12a) is a half-wave rectifier with a smoothing capacitor. The diode *D* is in charge of rectifying the AC signal with the DC component to twice the input voltage peak, and the capacitor *C* considerably reduces the output voltage ripple *δV*. The HWCW circuit and its operation principle are depicted in Figure 12. The greater the number of stages *N* placed in the circuit, the higher the output voltage is [23].

Two circuit elements constitute a HWCW multiplier circuit. The diode is the most critical element, as it has to switch at frequencies of GHz and has to consume a very reduced amount of power. The rectifying efficiency of the multiplier depends mostly on the forward voltage drop $V_{F}$ of the diode. Since there are two diodes per stage, the output voltage reduces by $2NV_{F}$ from the ideal value. Thus, it is preferable to use a single stage when the input power is low. A Schottky diode very suitable for this situation is the HSMS-2822 [28]. The HSMS-2822 package contains two series HSMS-2820 diodes, specifically designed for low power operation at frequencies below 4 GHz. According to the datasheet [28], the HSMS-2822 diodes can provide currents of 0.1 mA with a voltage drop of 0.22 V (@ 25 °C). The capacitor must be chosen in order to reduce the output ripple. The higher the capacitance value is, the lower the voltage ripple at the output. However, the parasitics of the capacitor play an important role. These are related with the concept of self-resonant frequency (SRF). The higher the SRF, the lower the effect of the parasitics in the circuit is. The parasitic of a capacitor is modeled as a series parasitic inductance, while the parasitic of an inductor is modeled as a shunt capacitor. Both are LC circuits, so the SRF is

(2) $$SRF\;\left[\mathrm{Hz}\right]=\frac{1}{2\pi \sqrt{LC}}$$

Thus, the parasitic value can be calculated by measuring the SRF in the laboratory and using Equation (2). In [29], a 33 pF capacitor is used as a tradeoff value between the output ripple and the SRF.

### 4.1. Design Procedure

As already discussed in Section 1, the matching circuit is primarily responsible for maximizing the power transfer from the antenna to the conditioning circuit. However, since the half-wave Cockcroft-Walton multiplier has a strong non-linear behavior, the design of the matching circuit can become a complicated task. The multiplier circuit is highly sensitive to slight variations in the input power, frequency and other terms. Specifically, the design of the matching circuit depends of five parameters: input power, input frequency, antenna impedance, load (type of sensor), and the number of stages *N* in the HWCW multiplier. Fortunately, the input power and the input frequencies can be estimated according to the power spectrum acquired by the Archimedean spiral antenna. We also know that the antenna impedance has a theoretical value of 188 Ω. Thus, the complexity of the design can be reduced to only two dependent terms: the number of stages *N* in the multiplier circuit and the load impedance. The design of the matching circuit is as follows. The antenna impedance is replaced by a series equivalent impedance $Z_{s}$ placed after the generator.

**Figure 13:**
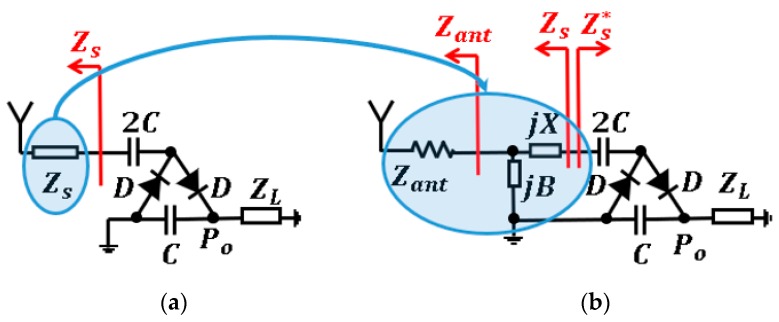
Design procedure of the matching circuit. Obtaining the optimum source impedance $Z_{s}$ (**a**) and implementing the matching circuit (**b**).

The value of $Z_{s}$ that maximizes the output power $P_{o}$ is then sought via an optimization process. Power transfer is then maximized when the matching circuit is able to transform the antenna impedance $Z_{ant}$ (188 Ω) into the optimum equivalent impedance $Z_{s}$. Subsequently, the components that conform the matching circuit (*L* matching network) are determined in order to provide the desired impedance transformation. The design procedure for a single stage of the HWCW multiplier is clearly depicted in Figure 13.

### 4.2. Test Circuit. Modeling the Parasitics

There are several ways of designing a lossless matching circuit [29]. The vast majority of them are based on the use of transmission lines and stubs, or on the use of lumped elements. The use of transmission lines is prohibitive in our particular case due to the low frequency involved in the design (800 MHz) and, therefore, the large dimensions of the lines. Thus, we focus our study on the use of lumped elements. The simplest matching network of this kind is the *L* section [29]. It is formed by two reactive elements and is capable of transforming a real impedance (188 Ω) into a certain complex load within the required narrow band (from 800 MHz to 950 MHz).

An accurate circuit model is needed, capable of predicting the measurement deviations caused by the parasitic elements present in the components and in the PCB itself. The creation of the circuit model follows these steps: firstly, the parasitic components of inductors and capacitors are estimated in the laboratory via their self-resonant frequency. They are related according to Equation (2). The SRF of a given inductor or capacitor is obtained in the laboratory by placing the component in series with a 50 Ω line [30]. The zero reflection indicates the SRF of the capacitor, while the zero transmission shows the SRF of the inductor. Finally, the rest of the parameters (parasitic inductance of the via hole and parasitic shunt capacitance of the HSMS-2822 package) are tuned in the simulator on the basis of theoretical values [30].

**Figure 14:**
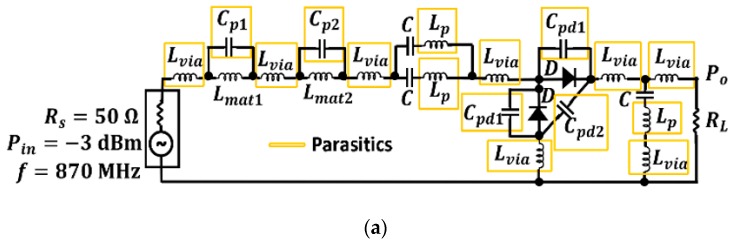

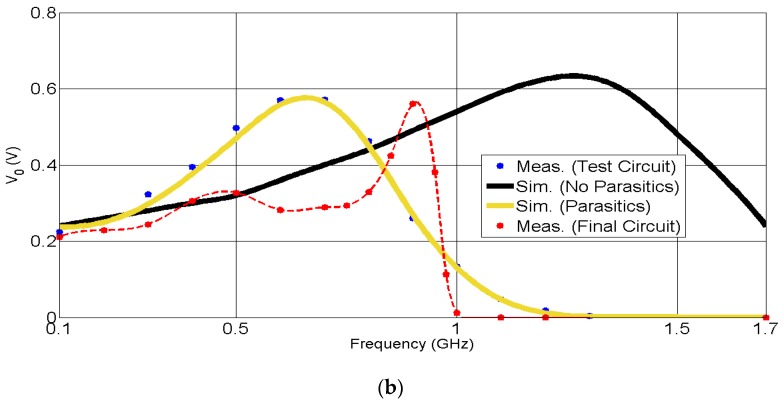
Test circuit with the parasitic elements modeling (**a**), and a comparison between measurement and simulation (**b**). The measurement of the response in the final circuit appears marked in red.

The circuit model presented in Figure 14a includes four different types of parasitics: two of them are the parasitic capacitances and inductances associated with inductors $L_{mat1}=4.3\;\mathrm{nH}$, $L_{mat2}=8.2\;\mathrm{nH}$ and capacitor C=33 pF, respectively. Here, it can be found that $C_{p1}=0.10\;\mathrm{pF}$, $C_{p2}=0.19\;\mathrm{pF}$, and $L_{p}=0.16\;\mathrm{nH}$. The third type of parasitic models the effect of via holes in the circuit by means of the parasitic inductance $L_{via}=1.30\;\mathrm{Nh}$. The fourth type models the parasitic capacitance associated with the diode package through $C_{pd1}=0.75$ pF, and $C_{p2}=0.10$ pF. It is determined that the most harmful parasitic elements in the circuit model are those related to the inductors and the diodes. That is, $C_{pd1},\;C_{p1}\;\mathrm{and}\;C_{p2}$. On the other hand, the parasitic inductance $L_{p}$ and the parasitic capacitance $C_{p2}$ are completely negligible [30].

A comparison between the measurement of the test circuit, a simulation where the parasitics have not been considered, and a simulation with the parasitics are properly modeled is presented in Figure 14b. The parasitic elements considerably displace the resonance peak from the basic simulation (black line) to the measurement (blue dots). Therefore, it is necessary to model the parasitics of each designed circuit. By doing so, the agreement between the measurement and the simulation with the parasitic elements (yellow line) is noticeable.

### 4.3. Final Circuit. Efficiencies

**Figure 15:**
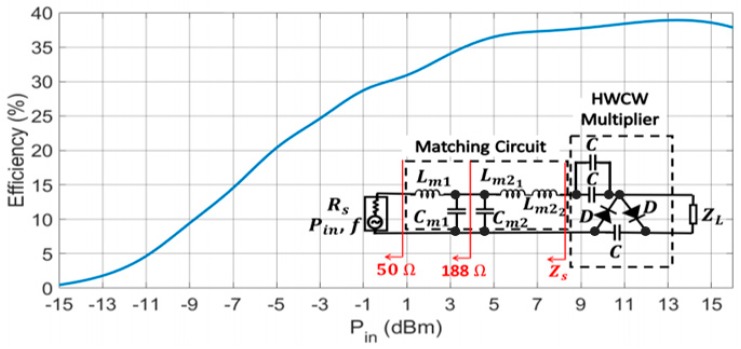
Measured efficiency as a function of the input power in the final circuit (at 870 MHz).

The correct modeling of the parasitics in the test circuit serves as the basis for the design of the final circuit, whose schematic is presented in Figure 15. Thus, the best performance of the final circuit is situated in the 800–900 MHz band. This circuit is formed by a single stage of the HWCW multiplier (two capacitors are placed in parallel to reduce charge losses [23]) and two *L* matching sections. The first of them is expendable and must be eliminated when the antenna is physically connected to the circuit. It transforms the inner impedance of the generator (50 Ω) into the impedance of the antenna (188 Ω) by means of $L_{m1}=7.5$ nH and $C_{m1}=1.5$ pF. Otherwise, the circuit could not be measured with a 50 Ω signal generator. The second *L* section is formed by $C_{m2}=4.7$ pF, $L_{m2_{1}}=8.2$ nH, and $L_{m2_{2}}=4.3$ nH. It transforms the antenna impedance into the optimum source impedance $Z_{s}$. This section is actually in charge of maximizing the power transfer between the antenna and the multiplier circuit in the frequency bands of 800 MHz and 900 MHz.

Figure 15 shows the measured efficiency in the final circuit as a function of the input power. The 870 MHz input tone is provided by an Agilent E4438C vector signal generator. Simulating the effect of the sensor, a load of value $Z_{L}=8$ kΩ has been placed. If we consider the minimum activation power as the value where the efficiency drops to zero in Figure 15, the multiplier circuit starts to work from −15 dBm onwards. For the harvested power levels, close to 0 dBm, the rectifying efficiency is 30%. A drop in the efficiency of approximately a 7% is also observed when the input power is reduced by half (−3 dBm) from 5 dBm downwards. On the other hand, the efficiency saturates from 10 dBm onwards. A drop in the efficiency can even be observed above 15 dBm, which is caused by the diodes. They are specifically designed to operate within a small signal region, so their performance is degraded above this input power.

### 4.4. Storage Capacitor

**Figure 16:**
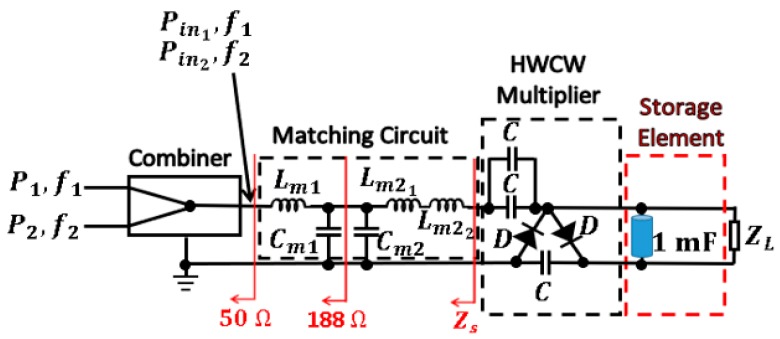
Setup used to obtain the charge and discharge curves of the 1 mF storage capacitor.

**Figure 17:**
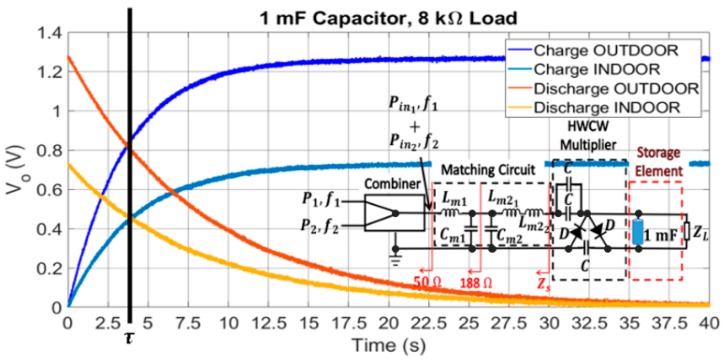
Charge and discharge curves of a 1 mF capacitor when using an 8 kΩ resistive load.

Charge and discharge curves of a certain storage capacitor can be used as a preliminary test to detect whether the circuit is able to correctly operate as a DC power supply. Thus, a 1 mF capacitor is placed in parallel to the 8 kΩ load. As shown in Figure 16, the setup used to characterize the charge and discharge curves in the capacitor is formed by two signal generators, a microwave combiner, and the circuit itself. The function of the combiner is to generate an input waveform for the circuit, which is the sum of two tones centered at 807 MHz and 942 MHz. Thus, the circuit model of the Archimedean spiral antenna presented in Section 3.4 can be replicated. Figure 17 presents the charge and discharge curves for the 1 mF capacitor. The capacitor charges to 1.30 V and 0.80 V in the outdoor and indoor scenarios, respectively, with the characteristic exponential curve of a RC circuit. The time constant τ of the circuit is found to be the same in both scenarios, τ = 3.8 s. This is the required time to charge the capacitor to the 63.2% (charge curve) of its steady voltage value (1.30 V and 0.80 V). This is also the cross point between the charge and discharge curves for each scenario, as seen in Figure 17. Note that the time constant is almost half of the load resistance times the value of the storage capacitor, 8 kΩ × 1 mF. This means that the output source resistance of the DC power supply that forms the combiner, the *L* matching section, and the HWCW multiplier is almost 8 kΩ. To be more specific, 8.84 kΩ, according to τ = 3.8 s. Therefore, it is indirectly seen that the power transfer is nearly optimal. It would be really optimal if the time constant of the whole circuit were 4 s (half of the load resistance times the storage capacitor) instead of τ = 3.8 s.

### 4.5. Temperature and Humidity Sensor

Once the whole circuit has been demonstrated to operate as a DC power supply, the 8 kΩ load is replaced by a specific sensor. This sensor displays the temperature and the relative air humidity in an LCD screen, which needs to be biased over 1.25 V to be switched on. It consumes currents between 50 μA and 80 μA, depending on the sensor requirements. For instance, the power consumption will be higher when the digits of the LCD screen are being updated. Therefore, the sensor can be seen as a dynamic load at the circuit level, with an impedance that varies from 20 kΩ to 30 kΩ.

**Figure 18:**
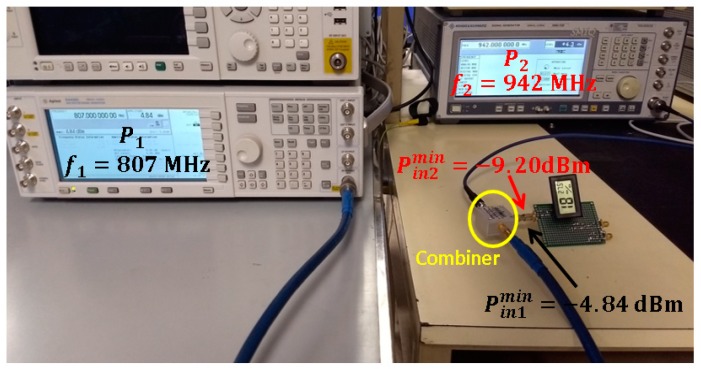
Measurement setup for the characterization of the minimum powers that turn on the temperature sensor.

Several combinations of input powers $P_{1}$ and $P_{2}$ are attempted in the combiner in order to determine the minimum power $P_{in_{1}}$ and $P_{in_{2}}$ that turns on the sensor. They are found to be ${P_{in}}_{1}^{min}\;=-4.84$ dBm and ${P_{in}}_{2}^{min}=-9.20$ dBm. Figure 18 depicts the measurement setup. Please note that the power measurement in the outdoor scenario ($P_{{in}_{1}}\;=-1.46$ dBm and $P_{{in}_{2}}\;=-2.34$ dBm, shown in Section 3.4) exceeds the minimum required power, which enables the circuit to work properly under this condition. However, the power level acquired in the indoor scenario ($P_{{in}_{1}}=-5.80$ dBm and $P_{{in}_{2}}=-9.20$ dBm, shown in Section 3.4) is not sufficient to turn on the sensor.

Then, the autonomy of the circuit is tested in the laboratory. For the power requirement in the outdoor scenario, it is observed that the storage capacitor maintains operation of the temperature sensor for 87 s after the circuit is disconnected from the signal generator.

## 5. Full-System Validation

**Figure 19:**
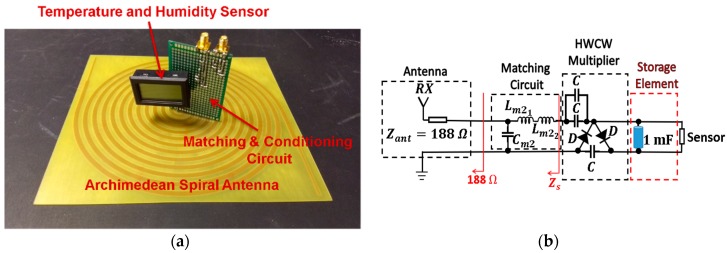
Full RF harvesting system (**a**) and its circuit diagram (**b**). $C_{m2}=4.7$ pF, $L_{m2_{1}}=8.2$ nH, $L_{m2_{2}}=4.3$ nH, C=33 pF.

The full RF energy harvesting system and its circuit diagram are shown in Figure 19. The arms of the Archimedean spiral antenna are welded to the positive and the negative poles of the circuit to maintain a balanced structure. As discussed in Section 4.2, the first *L* matching section shown in the circuit diagram of Figure 15 and Figure 16 has been eliminated. The antenna already acts as 188 Ω of resistance, so the matching section that transforms 50 Ω into 188 Ω is no longer needed.

**Figure 20:**
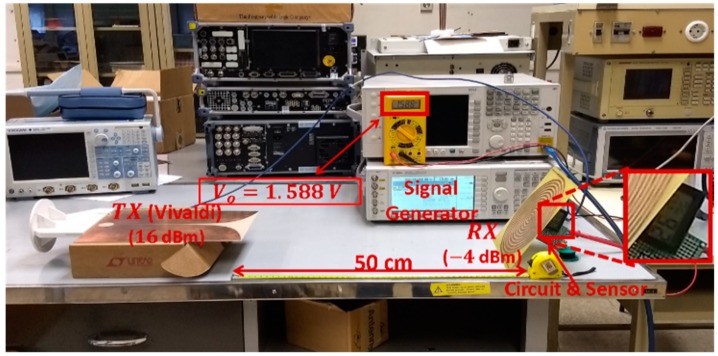
Full-system validation: receiving RF energy harvesting system (on the right) in front of a transmitting Vivaldi antenna (on the left).

The operation of the whole system is then tested in the laboratory. For this purpose, the RF harvesting system is placed at a distance of 50 cm from a transmitting source, as depicted in Figure 20. A signal generator injects a 16 dBm tone, centered at 807 MHz, on a wideband transmitting Vivaldi antenna. Then, the spiral collects the incoming power and the circuit attached to the antenna conditions the acquired power in order to supply the temperature sensor. A multimeter located in the center of the figure shows the voltage in the sensor. A rough link budget analysis is carried out to determine the power level that reaches the Archimedean spiral. The gains of the Vivaldi and the spiral antenna are 4.75 dB and 3 dB, respectively. Depolarization losses (3 dB) must be taken into account, since the Vivaldi antenna is linearly polarized and the spiral antenna is circularly polarized. Both antennas are separated by 50 cm, and the transmitting frequency is 807 MHz, so free space path loss is 24.56 dB. If misalignment antenna loss is neglected, the link budget analysis shows that the incoming power on the spiral antenna is approximately −4 dBm. This amount of power is enough to charge the 1 mF capacitor and to turn on the temperature sensor. In fact, the sensor actually displays on the LCD screen a relative humidity of 26% and a temperature of 25.5 °C (77.9 °F) inside the laboratory. As seen in Figure 20, the multimeter reveals that the voltage in the sensor is 1.588 V, which exceeds the required 1.25 V to turn on the sensor.

Finally, a comparison among different state-of-the-art RF energy harvesting systems is presented in Table 1. As can be noticed, most of the implemented solutions make use of narrowband antennas that work at WiFi/Bluetooth frequency band (2.4 GHz). However, it has already been discussed in the manuscript that they have the disadvantage of not being interoperable among countries with different frequency assignment plans. The rectifying efficiency of our circuit is comparable with the state-of-the-art designs [5,8,13,14,31], and even higher in some cases. However, unlike all the other references presented in Table 1, our system has been additionally tested with a temperature sensor acting as a load. It has been proved that the sensor is perfectly operational if the antenna is able to collect at least −4 dBm. This is a further step in order to corroborate the fine operation of the RF energy harvesting system and demonstrates its validity for practical applications.

## 6. Conclusions

This paper presents the design, simulation, manufacture and measurement of a RF energy harvesting system based on an Archimedean spiral antenna and a half-wave Cockcroft-Walton multiplier. The main purpose of this work is to present a deep study of a complete RF harvesting system, paying attention to each individual block and to the entire system, deeply analyzing all the parameters, factors and parasitic effects that have influence in the system, aiming towards its optimization. The miniaturized spiral antenna operates from 350 MHz to 16 GHz, showing an ultrawideband behavior. With its use, it is determined that 98% of the harvested power comes from the mobile frequency bands. In particular, 82% of the total power belongs to 800 MHz and 900 MHz frequency bands. Therefore, the antenna is modeled at a circuit level as two series sinusoidal generators, centered at 807 MHz and 942 MHz. It is also observed that a greater amount of power is captured with the antenna outdoors than indoors. The parasitic elements of the board and the components play a crucial role in the design of the matching and conditioning circuits. With its corresponding modeling, a circuit is designed whose rectifying efficiency is 30% for an input power of 0 dBm. Afterwards, a storage capacitor is placed in parallel with a temperature sensor to test the performance of the circuit. By means of the study of the charge and discharge curves in the storage capacitor, it is proved that the circuit is able to act as a DC power supply.

Finally, the Archimedean spiral is physically attached to the circuit. A test is carried out in the laboratory to check the correct performance of the full system. For this purpose, the RF harvesting system is placed in front of a transmitting Vivaldi antenna at a distance of 50 cm. The storage capacitor charges over 1.25 V, so the sensor correctly displays the temperature and the relative air humidity on the LCD screen. This test demonstrates the fine operation of the full RF energy harvesting system and shows its capabilities for use in practical applications.

## Figures and Tables

**Table 1:** Comparison among different RF energy harvesting systems.

Ref.	Antenna (Freq.)	Circuit (Freq.)	Circuit Efficiency (Input Power)	Sensor Testing
[5]	Array of Log. Spiral (2–18 GHz)	Half-Wave Rectifier (-)	20% (4 dBm)	NO
[8]	Linear Tapered Slot Antenna (1.85/2.4 GHz)	Differential Rectifier (1.85 GHz)	13% (−15 dBm)	NO
[13]	Concentric Square Patches (2.4/5.5 GHz)	Full-Wave Rectifier (2.4/5.5 GHz)	36% @2.4 GHz 5% @5.5 GHz (0 dBm)	NO
[14]	Slotted Patch Antenna (2.45 GHz)	Half-Wave Cockcroft-Walton (2.45 GHz)	68% (5 dBm)	NO
[31]	Meander Antenna (434 MHz)	Voltage Doubler (434 MHz)	20% (−30 dBm)	NO
**This Work**	Arch. spiral (0.3–16 GHz)	Half-Wave Cockcroft-Walton (800/900 MHz)	30% (0 dBm)	Temperature Sensor
